# Therapeutic efficacy and prognostic indicators in re-resection for recurrent hepatocellular carcinoma: Insights from a retrospective study

**DOI:** 10.1016/j.sopen.2024.12.004

**Published:** 2024-12-18

**Authors:** Qi Fan, Pengcheng Wei, Delin Ma, Qian Cheng, Jie Gao, Jiye Zhu, Zhao Li

**Affiliations:** a Department of General Surgery, Beijing Jishuitan Hospital, Capital Medical University, Beijing, China; b Department of Hepatobiliary Surgery, Peking University People's Hospital, Beijing, China; c Beijing Key Surgical Basic Research Laboratory of Liver Cirrhosis and Liver Cancer, Beijing, China; d Peking University Center of Liver Cancer Diagnosis and Treatment, Beijing, China; e Peking University Institute of Organ Transplantation, Beijing, China

**Keywords:** Recurrent hepatocellular carcinoma, *Re*-resection, Prognosis, Survival analysis, Immune microenvironment

## Abstract

**Aims:**

To evaluate the efficacy of re-resection in recurrent hepatocellular carcinoma (rHCC), identify prognostic factors, and provide clinical guidance.

**Methods:**

A retrospective analysis was conducted on 130 rHCC patients undergoing re-resection and 60 primary HCC patients undergoing initial hepatectomy at Peking University People's Hospital (2014–2022). Disease-free survival (DFS) and overall survival (OS) were compared. Prognostic factors were identified using univariate and multivariate COX regression analyses.

**Results:**

Baseline characteristics were comparable between groups (*P* > 0.05). DFS was similar between groups (30.8 vs. 32.2 months, *P* = 0.612). The 1-year, 2-year, and 3-year DFS rates for the re-resection group were 88.5 %, 64.9 %, and 56.7 %, respectively, versus 88.3 %, 65.0 %, and 53.3 % for the primary resection group. OS was lower in the re-resection group (36.1 vs. 47.2 months, *P* = 0.041) with 1-year, 2-year, and 3-year OS rates of 90.8 %, 73.1 %, and 60.0 %, compared to 95.0 %, 80.0 %, and 68.3 % for the primary resection group. Significant factors affecting DFS were Child-Pugh classification (*P* = 0.044), time to recurrence (*P* = 0.002), tumor differentiation (P = 0.044), and satellite nodules (*P* = 0.019). Factors influencing OS included Child-Pugh classification (*P* = 0.040), time to recurrence (*P* = 0.002), and tumor differentiation (*P* = 0.032).

**Conclusions:**

*Re*-resection is an effective treatment option for rHCC, with favorable outcomes as measured by DFS and OS, though OS is lower compared to initial hepatectomy. Key prognostic factors include Child-Pugh classification, time to recurrence, tumor differentiation, and satellite nodules.

## Introduction

Hepatocellular carcinoma (HCC) is the sixth most common malignant tumor worldwide and the third leading cause of cancer-related death [1]. In China, it is the second leading cause of cancer-related death, with a five-year overall survival (OS) rate of <20 % [2,3]. HCC has an insidious onset, rapid progression, and is closely associated with chronic liver diseases such as hepatitis B and C. Most patients are diagnosed at intermediate to advanced stages [4,5]. Despite the five-year survival rate reaching 50 % after curative resection of primary HCC, the recurrence rate remains as high as 60–70 %, significantly limiting long-term outcomes [5,6].

Recurrent HCC (rHCC) exhibits more aggressive tumor biology and is more prone to recurrence and metastasis than primary HCC. Patients with rHCC typically have poor health status and decreased liver functional reserve [7,8]. Based on clonal origin, rHCC can be classified into intrahepatic metastasis (IM) and multicentric occurrence (MO), which differ significantly in biological behavior and clinical management [9]. MO-type rHCC is associated with a better prognosis and is more suitable for curative treatments such as re-resection or salvage liver transplantation (SLT), whereas IM-type rHCC generally has a worse prognosis and responds better to transarterial chemoembolization (TACE) or systemic therapy [10,11].

In recent years, advancements in immune-based therapies and molecular classification have added complexity to treatment decision-making for rHCC. However, the indications and contraindications for re-resection remain controversial. While studies have demonstrated its safety and efficacy, high-level evidence to support the role of re-resection in rHCC is still lacking [[12], [13], [14]].

This study retrospectively analyzed clinical cases of rHCC patients undergoing re-resection at our center, comparing them to cases of primary HCC resection during the same period. The objectives were to evaluate the therapeutic efficacy of re-resection, identify key prognostic factors, and provide evidence-based guidance for clinical decision-making, aiming to address gaps in the current literature on rHCC management.

## Methods

### Patients

We collected clinical and pathological data from 130 patients who underwent re-resection for rHCC at the Department of Hepatobiliary Surgery, Peking University People's Hospital, between January 2014 and December 2022. Additionally, data from 60 primary HCC cases who underwent initial resection by the same surgical team during the same period were analyzed. All surgeries were performed by six senior hepatobiliary surgeons, each with over 10 years of experience and an average of >300 liver resections completed. To ensure procedural consistency, all operations followed a standardized protocol established by the department, covering preoperative assessment, application of anatomic liver resection techniques, and postoperative management. Surgical plans for all cases were reviewed and approved during multidisciplinary team (MDT) meetings, involving experts from hepatobiliary surgery, oncology, radiology, and pathology. This approach minimized selection bias and ensured standardized surgical decision-making. The decision-making process was further guided by international guidelines (e.g., NCCN and EASL guidelines) and the clinical expertise of our center, incorporating individualized considerations for each patient to minimize selection bias and ensure consistency and reliability in patient inclusion criteria.

Inclusion criteria included curative liver resection with no residual tumor, pathologically confirmed HCC, no recurrence within one month postoperatively, Child-Pugh classification A or B, no lymph node or distant metastasis, and Eastern Cooperative Oncology Group (ECOG) performance status score of 0–1. Limiting inclusion to Child-Pugh A/B and ECOG 0–1 patients aimed to assess re-resection efficacy in those with better liver function and performance status, minimizing confounding factors. Additionally, selection criteria in MDT meetings considered recurrence type (e.g., intrahepatic metastasis [IM] vs. multicentric occurrence [MO]), lesion characteristics (tumor size, number, vascular invasion), and liver function assessment. Exclusion criteria included loss to follow-up after surgery, presence of other malignancies, severe organ dysfunction (e.g., heart, brain, lung), and death within one month postoperatively. Patients who died within one month were excluded to avoid the confounding effect of perioperative mortality on long-term survival analysis. Loss to follow-up was defined as the inability to collect survival or recurrence data through telephone interviews or outpatient visits. This study was conducted in accordance with the principles outlined in the Declaration of Helsinki. Ethical approval was obtained from the Institutional Review Board of Peking University People's Hospital (Approval No. 2022PHB092–001). Written informed consent was obtained from all participants prior to surgery, allowing the use of their clinical specimens for scientific research.

### Data collection

We reviewed electronic medical records to collect patient information, including gender, age, preoperative levels of alpha-fetoprotein (AFP), total bilirubin (TBiL), alanine aminotransferase (ALT), and aspartate aminotransferase (AST); presence of preoperative cirrhosis; concurrent hepatitis B virus (HBV) infection; Child-Pugh classification; time to recurrence (≥24 months vs. <24 months); number of recurrent tumors (single vs. multiple); size of recurrent tumors; microvascular invasion; satellite lesions; and tumor differentiation grade. Recurrence was diagnosed based on radiological evidence (CT or MRI) of new or progressive lesions, with pathological confirmation where necessary. Time to recurrence was defined as the interval between the re-resection and the confirmation of recurrence by imaging or pathology. Recurrence occurring within 24 months postoperatively was classified as early recurrence, while recurrence after 24 months was classified as late recurrence.

Postoperative follow-up was conducted through outpatient visits or telephone interviews to monitor recurrence and survival status. Follow-up endpoints included disease-free survival (DFS) and overall survival (OS). DFS was defined as the time from re-resection to the diagnosis of recurrence or metastasis, or to the end of follow-up. OS was defined as the time from re-resection to death from HCC or other causes, or to the end of follow-up. The last follow-up was conducted in January 2024.

### Statistical analysis

All statistical analyses were conducted using SPSS version 24.0 (SPSS Inc., Chicago, IL, USA) and R software version 4.2.1. Data types included count and continuous data. Normality of continuous data was tested using the Kolmogorov-Smirnov test. Normally distributed data were described as mean ± standard deviation ($\bar{X}$ ±S) and analyzed using *t*-tests. Non-normally distributed data were described as median and interquartile range (M[P25-P75]) and analyzed using Mann-Whitney *U* tests. Count data were described using counts and percentages, with chi-square tests used for between-group analysis. Survival times were analyzed using the Kaplan-Meier method. Prognostic factors were assessed using the Log-rank test for univariate analysis and the COX proportional hazards regression model for multivariate analysis. Hazard Ratios (HR) were used to describe inter-group survival differences. R software with the survival (3.3.1) package was used for proportional hazards testing and Cox regression analysis, while the rms (6.3.0) package was used for constructing nomograms. The efficiency of the nomogram was validated using the concordance index, calibration curve, and decision curve analysis. A two-sided *P* value of <0.05 was considered statistically significant.

### Genetic tests

Genetic tests were conducted on 16 resection group cases that met quality standards, analyzing 29 common gene loci, including TP53, LRP1B, TERT, ATM, AXIN1, CCND1, CREBBP, EPHA5, AR, CRLF2, EPHA3, EPHB1, FANCD2, FAT3, FGF19, FGF3, FGF4, FGFR1, GRM3, IRS2, KEAP1, KMT2C, KMT2D, NOTCH1, NTRK2, PRKDC, PTPRT, SETD2, and TET2, to identify survival-impacting mutations. Immune microenvironment markers were assessed in suitable resection group samples using multiplex fluorescence immunohistochemistry. We analyzed eight markers: CD8 (cytotoxic T lymphocytes), CD3 (general T lymphocytes), CD68 (macrophages), CD163 (M2 macrophages), CD56 (natural killer cells), PD-L1 (programmed death receptor ligand 1), PD-1 (programmed death receptor 1), and panCK (epithelial-derived tumors). These markers were used to characterize expression profiles and quantify immune cell populations.

## Results

### Baseline characteristics of included patients

This study included 130 cases in the re-resection (RR) group, consisting of 104 males (80.0 %) and 26 females (20.0 %), with an average age of 60.3 ± 9.3 years. The primary resection (PR) group consisted of 60 cases, with 47 males (78.3 %) and 13 females (21.7 %), and an average age of 62.6 ± 8.6 years. Comparative analysis of baseline characteristics between the RR and PR groups showed no statistically significant differences (*P* > 0.05) in gender, age, preoperative AFP levels, preoperative tBIL levels, preoperative AST and ALT levels, presence of cirrhosis, HBV infection, preoperative Child-Pugh classification, time to recurrence, number of tumors, presence of intravascular tumor thrombus, satellite nodules, and tumor differentiation (Table 1).

**Table 1:** Baseline characteristics of RR and PR group^a^.

Variables	RR	PR	F/*χ*^*2*^	*P* value
Sex [n (%)]			0.070	0.791
Male	104 (80.0 %)	47 (78.3 %)		
Female	26 (20.0 %)	13 (21.7 %)		
Age (years)	60.3 ± 9.3	62.6 ± 8.6	0.877	0.682
Pre-operative AFP (ug/mL)	8.3 (3.5–100.6)	11.7 (5.6–49.6)	1.022	0.307
Pre-operative TBiL (μmol/L)	15.6 ± 7.0	15.9 ± 4.7	0.350	0.727
Pre-operative AST (U/L)	38.3 ± 37.6	38.9 ± 21.2	0.112	0.911
Pre-operative ALT (U/L)	46.9 ± 26.7	48.3 ± 28.2	1.309	0.428
Cirrhosis [(n%)]			0.632	0.562
Yes	79 (60.8 %)	43 (71.7 %)		
No	51 (39.2 %)	17 (28.3 %)		
HBV infection [(n%)]			0.342	0.559
Yes	94 (82.3 %)	42 (70.0)		
No	36 (27.7 %)	18 (30 %)		
Child-Pugh classification [(n%)]			0.246	0.540
A	102 (78.5 %)	39 (65.0 %)		
B	28 (21.5 %)	21 (35.0 %)		
Tumor size (cm)	4.1 ± 2.1	4.7 ± 2.8	4.439	0.056
Number of tumors [(n%)]			0.788	0.375
Single	87 (67.0 %)	44 (73.3 %)		
Multiple	43 (33.0 %)	16 (16.7 %)		
Presence of intravascular tumor thrombus [(n%)]			2.023	0.155
Yes	68 (52.3 %)	38 (63.3 %)		
No	62 (47.7 %)	22 (16.7 %)		
Presence of satellite nodules [(n%)]			0.091	0.762
Yes	32 (24.6 %)	16 (26.7 %)		
No	98 (75.4 %)	44 (75.3 %)		
Tumor differentiation grade [(n%)]			2.293	0.318
Well	26 (20.0 %)	15 (25.0 %)		
Moderate	78 (60.0 %)	29 (48.3 %)		
Poor	26 (20.0 %)	16 (26.7 %)		

a RR: re-resection; PR: primary resection; AFP: alpha-fetoprotein; TBiL: total bilirubin; AST: aspartate aminotransferase; ALT: alanine aminotransferase.

### Prognostic comparison: re-resection vs. primary resection

**Fig. 1:**
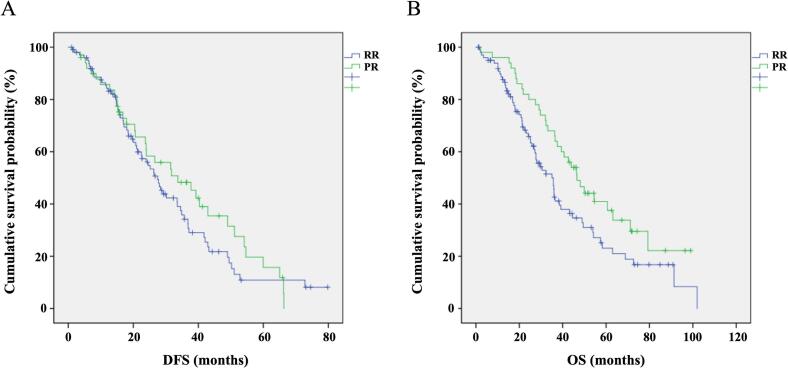
K-M survival curve between RR and PR. A. Comparison of DFS between RR and PR; B. Comparison of OS between RR and PR.

Kaplan-Meier survival analysis was performed on 130 patients who underwent re-resection for recurrent liver cancer and 60 patients who underwent primary liver resection. The re-resection group had 1-year, 2-year, and 3-year DFS rates of 88.5 %, 64.9 %, and 56.7 %, respectively, compared to 88.3 %, 65.0 %, and 53.3 % in the primary resection group. The median DFS was 30.8 months (95 % CI: 25.3–33.9) for the re-resection group and 32.2 months (95 % CI: 27.8–41.6) for the primary resection group, with no statistically significant difference (*P* = 0.612). The 1-year, 2-year, and 3-year OS rates in the re-resection group were 90.8 %, 73.1 %, and 60.0 %, respectively, while in the primary resection group, the rates were 95.0 %, 80.0 %, and 68.3 %. The median OS was 36.1 months (95 % CI: 31.9–39.5) for the re-resection group, significantly lower than the 47.2 months (95 % CI: 35.4–57.7) observed in the primary resection group (*P* = 0.041), as shown in Fig. 1.

The recurrence rates for the re-resection group were 11.5 %, 35.1 %, and 43.3 % at 1, 2, and 3 years, respectively. In the primary resection group, the recurrence rates were 11.7 %, 35.0 %, and 46.7 % at 1, 2, and 3 years, respectively. These results indicate a comparable recurrence pattern between the two groups over time, with a slightly higher recurrence rate observed in the primary resection group at 3 years.

### Analysis of factors affecting survival after re-resection

Initially, a univariate analysis was conducted to assess the impact of various factors on postoperative DFS and OS in the re-resection group. Independent variables included age, gender, preoperative serum AFP levels, TBiL levels, AST levels, ALT levels, presence of liver cirrhosis, HBV infection, Child-Pugh classification, tumor multiplicity, maximum tumor diameter, interval to recurrence, presence of satellite nodules, microvascular invasion, and tumor differentiation. The analysis identified significant predictors of DFS: time to recurrence (*P* = 0.003), presence of satellite nodules (*P* = 0.020), microvascular invasion (P = 0.020), and tumor differentiation (*P* = 0.027). Significant predictors of OS included preoperative AFP levels (*P* = 0.048), Child-Pugh classification (*P* = 0.029), time to recurrence (*P* = 0.001), microvascular invasion (*P* = 0.011), and tumor differentiation (*P* = 0.013). Detailed results are presented in Table 2.

**Table 2:** Univariate analysis of factors affecting DFS and OS after re-resection^a^.

Variables	Median DFS (95%CI)	χ^2^	*P* value	Median OS (95%CI)	χ^2^	*P* value
Age		0.713	0.386		0.839	0.581
≤60 years	27.5 (21.7–33.2)			36.0 (31.8–40.1)		
>60 years	25.3 (17.6–32.2)			29.4 (18.2–37.0)		
Sex		0.101	0.781		1.325	0.674
Male	27.9 (21.6–33.4)			33.7 (27.2–44.1)		
Female	27.8 (16.3–39.2)			34.2 (22.2–46.7)		
Pre-operative AFP		3.501	0.066		3.682	0.047
≤25 ng/mL	20.1 (11.9–28.2)			25.8 (15.5–33.5)		
>25 ng/mL	28.4 (21.5–35.3)			36.4 (28.8–44.3)		
Pre-operative TBiL		0.557	0.480		1.038	0.527
≤17.1 μmol/L	28.6 (22.9–33.0)			35.7 (29.5–41.9)		
>17.1 μmol/L	26.3 (20.9–31.1)			29.1 (14.0–42.0)		
Pre-operative AST		0.886	0.379		0.926	0.415
≤35 U/L	33.5 (19.6–48.0)			39.8 (14.7–64.1)		
>35 U/L	26.3 (19.0–33.9)			30.4 (26.5–43.1)		
HBV infection		0.471	0.660		2.721	0.112
Yes	26.8 (22.4–30.1)			33.5 (21.2–39.8)		
No	34.1 (12.6–49.7)			42.5 (31.6–54.9)		
Cirrhosis		2.572	0.108		0.082	0.789
Yes	25.6 (19.9–32.4)			32.1 (23.3–42.4)		
No	29.8 (18.3–40.8)			36.3 (24.7–46.6)		
Child-Pugh classification		3.647	0.059		4.790	0.029
A	31.8 (24.2–41.1)			39.1 (30.6–45.5)		
B	20.1 (11.6–31.7)			23.3 (16.8–28.4)		
Time to recurrence		8.629	0.003		10.204	0.001
<24 month	20.9 (15.8–25.9)			25.5 (21.0–29.7)		
≥24 month	35.7 (31.2–40.9)			48.8 (32.8–59.9)		
Number of tumors		2.813	0.086		1.572	0.281
Solid	32.2 (21.6–44.2)			36.4 (27.6–44,0)		
Multiple	25.7 (19.9–28.6)			28.9 (20.1–37.6)		
Tumor size		0.258	0.616		0.099	0.741
≤5 cm	28.0 (20.9–35.2)			35.7 (26.1–45.3)		
>5 cm	26.3 (22.1–30.6)			32.3 (22.2–42.3)		
Presence of intravascular tumor thrombus		5.392	0.022		2.380	0.097
Yes	22.8 (12.4–35.8)			28.3 (16.4–35.8)		
No	30.2 (20.9–39.1)			36.5 (28.2–44.1)		
Presence of satellite nodules		5.388	0.025		6.512	0.012
Yes	21.6 (11.2–33.7)			27.6 (16.7–38.6)		
No	31.2 (20.3–41.5)			39.7 (26.9–51.2)		
Tumor differentiation grade		7.231	0.019		7.939	0.011
Well	36.0 (24.3–46.1)			49.9 (24.4–64.8)		
Moderate	26.5 (21.8–31.2)			36.8 (29.0–49.3)		
Poor	12.8 (3.9–20.2)			15.7 (4.1–26.9)		

a DFS: disease-free survival; OS: overall survival; AFP: alpha-fetoprotein; TBiL: total bilirubin; AST: aspartate aminotransferase; HBV: hepatitis B virus.

Given the sample size constraints, variables with a *P*-value <0.1 in the univariate analysis were included in the subsequent Cox proportional hazards regression model for multivariate analysis. The Cox multivariate analysis revealed that Child-Pugh classification (*P* = 0.044), time to recurrence (*P* = 0.002), tumor differentiation (P = 0.044), and the presence of satellite nodules (*P* = 0.019) significantly influenced DFS. For OS, significant factors included Child-Pugh classification (*P* = 0.040), time to recurrence (P = 0.002), and tumor differentiation (*P* = 0.032), as shown in Table 3.

**Table 3:** Cox regression analysis of DFS and OS after re-resection^a^.

Variables	DFS		OS	
	HR	*P* value	HR	*P* value
Child-Pugh classification (A: B)	0.566	0.044	0.832	0.040
Time to recurrence (<24: ≥24 month)	2.248	0.002	2.305	0.002
Presence of satellite nodule (yes:no)	2.013	0.019		
Tumor differentiation grade (well: moderate: poor)	0.588	0.044	1.346	0.032

a DFS: disease-free survival; OS: overall survival.

### Nomogram for rHCC after re-resection

**Fig. 2:**
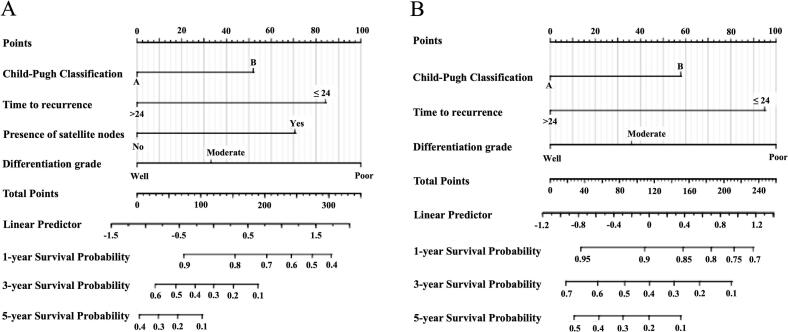
Prognostic nomogram for rHCC after re-resection. A. DFS Nomogram; B. OS Nomogram.

To enhance the visual assessment of prognostic factors for rHCC and identify patients who may benefit from re-resection, we developed nomograms for DFS and OS using significant predictors from the Cox regression analysis. These nomograms are shown in Fig. 2. The concordance index is 0.692 (95 % CI: 0.659–0.726) for the DFS nomogram and 0.698 (95 % CI: 0.660–0.737) for the OS nomogram.

### Genetic mutations in rHCC

**Fig. 3:**
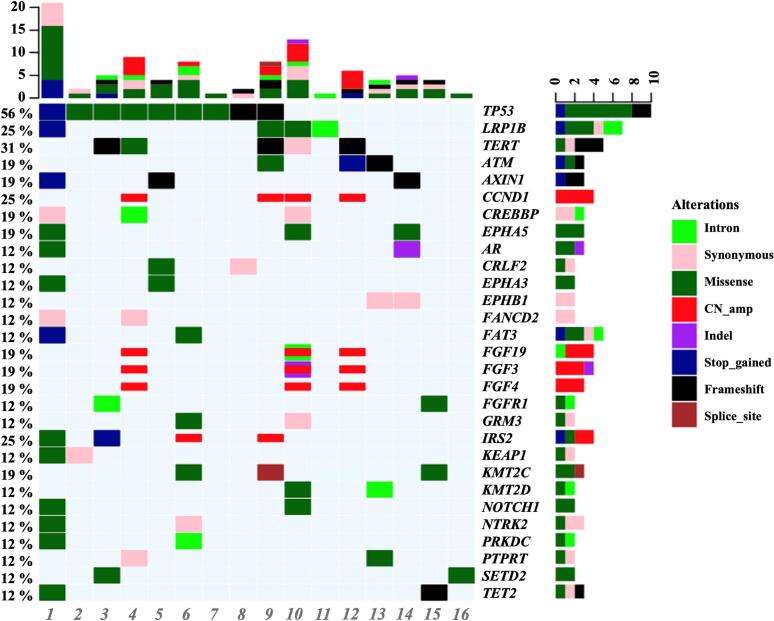
Gene mutations of 29 loci in rHCC.

Given the pronounced molecular heterogeneity in rHCC, our study aimed to delineate the genetic mutational landscape for precise classification. Among the 29 gene loci examined, TP53 exhibited the highest mutation frequency, as shown in Fig. 3. Both DFS and OS were shorter in the TP53 mutation group compared to the non-mutation group, though the differences were not statistically significant (DFS: *P* = 0.083, OS: *P* = 0.24).

### Differences in immune microenvironment between early and late recurrent lesions

**Fig. 4:**
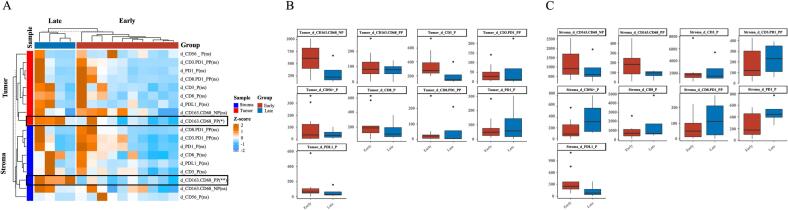
Immune microenvironment differences between early and late recurrence groups. A. Heatmap of immune microenvironment detection. B. Expression differences of immune markers in tumor specimens between early and late recurrence groups. C. Expression differences of immune markers in stromal specimens between early and late recurrence groups.

We meticulously screened rHCC specimens based on stringent quality standards for microenvironmental analysis, categorizing them into early recurrence (<24 months post-treatment) and late recurrence (24 months or later) groups. The analysis revealed significantly elevated expression levels of CD163 and CD68, markers of M2 macrophages, in the late recurrence group compared to the early recurrence group. This increase was consistent across both tumor specimens and associated stromal tissues, as shown in Fig. 4.

## Discussion

HCC is a major malignant tumor threatening human health, characterized by high mortality, high recurrence rate, and high heterogeneity, making clinical treatment challenging and prognosis poor [1,15,16]. Although the survival rate of HCC patients has significantly improved with advancements in modern medical technology, the high postoperative recurrence rate still results in unsatisfactory long-term outcomes. Surgical resection is well established as the most frequently applied radical treatment for primary HCC. However, its therapeutic efficacy in rHCC is still under investigation and lacks high-level evidence [14,17,18]. A study by Zou et al. involving 635 rHCC patients reported OS rates of 96.9 %, 74.8 %, and 47.8 % at 1, 3, and 5 years after re-resection, respectively [19]. A retrospective analysis by Li et al. involving 103 patients demonstrated that re-resection can achieve good clinical outcomes in rHCC patients who meet the indications, with a postoperative complication rate of 31.1 % and OS rates of 92.1 %, 78.2 %, and 54.4 % at 1, 2, and 5 years, respectively [20]. In this study, the 1-year, 2-year, and 3-year OS rates for the re-resection group were 90.8 %, 73.1 %, and 60.0 %, respectively, while the DFS rates were 88.5 %, 64.9 %, and 56.7 %, with corresponding recurrence rates of 11.5 %, 35.1 %, and 43.3 %, respectively. These results are consistent with previous reports, further confirming the effectiveness of re-resection for rHCC. However, despite favorable postoperative survival, the increasing recurrence rates highlight the importance of postoperative monitoring and further treatment for patients with recurrent HCC.

Patients with rHCC typically have more aggressive tumor characteristics and poorer liver function, making the choice of re-resection as a treatment option crucial. A study by Bednarsch et al. found that the median DFS and OS in the re-resection group were 29 and 41 months, respectively, which were not significantly different from survival after primary HCC surgery [21]. Our study similarly compared the DFS and OS of the re-resection and primary resection groups. The median DFS and OS of the re-resection group were 30.8 and 36.1 months, respectively, while those of the primary resection group were 32.2 and 47.2 months, respectively. The DFS was similar, but the OS was significantly different between the groups. Our findings are consistent with previous literature, emphasizing the inverse relationship between postoperative survival and the cumulative number of surgeries [22,23]. Nonetheless, this does not negate the safety and efficacy of re-resection as a treatment modality for rHCC.

Identifying factors that influence postoperative survival in rHCC is crucial for improving long-term patient prognosis. Although the definition of high-risk recurrent metastatic factors varies among studies, high-risk factors for postoperative evaluation typically include: tumor rupture, tumor diameter > 5 cm, multiple tumors, satellite nodes, microvascular invasion, macrovascular invasion, lymph node metastasis, high preoperative AFP levels, impaired liver function, positive or narrow margins, and Edmondson grade III-IV tissue differentiation. These factors often indicate a higher likelihood of HCC recurrence after surgery [15,[24], [25], [26]]. The time points traditionally used to define early and late recurrence are typically set at 24 months or 12 months post-surgery [27]. In this study, of the 130 rHCC patients, 71 had recurrence >24 months after re-resection, which we defined as late recurrence, while 59 patients had recurrence within 24 months, defined as early recurrence. Similar to previous studies, our findings also showed that early recurrence was associated with significantly poorer DFS (20.9 months vs. 35.7 months, *P* = 0.003) and OS (25.5 months vs. 48.8 months, *P* = 0.001). This association was further confirmed in multivariate Cox regression analysis, highlighting the significant link between early recurrence and worse survival outcomes.

The current consensus suggests that early recurrence of HCC is closely associated with intrahepatic metastases, while late recurrence predominantly manifests as a multicentric occurrence [28]. Early recurrence indicates higher malignant potential and invasive capacity of the tumor, contributing to poor prognosis. It also suggests that recurrent tumors are more likely to be molecularly consistent with the primary tumor, indicating a monoclonal origin. In contrast, late recurrences usually occur in the context of chronic liver disease, such as viral hepatitis, steatohepatitis, or cirrhosis. These recurrent tumors show high genetic diversity and have characteristics of “de novo” malignancy compared to the primary lesion. This genetic heterogeneity is usually associated with a relatively good prognosis, reflecting the complex interplay between recurrence time and the underlying hepatic environment [29]. In this study, we also evaluated the relationship between the characteristics of the primary tumor (e.g., size, grade, vascular invasion) and the prognosis of rHCC. However, preliminary analyses showed that these pathological variables had no significant impact on postoperative outcomes, possibly due to the limited sample size and insufficient statistical power. Therefore, these variables were not included in the survival analysis, and larger sample sizes may be needed to draw more reliable conclusions. Additionally, the results of the present study suggest that Child-Pugh classification, tumor differentiation degree, and the presence of satellite nodules are strongly correlated with the prognosis of rHCC undergoing re-resection. Lower tumor differentiation and the presence of satellite nodules indicate higher malignancy and greater capacity for invasion and metastasis. Child-Pugh classification reflects hepatic functional status. A poorer Child-Pugh classification indicates impaired hepatic reserve function, creating a more favorable environment for tumor growth. These factors have been shown to correlate with a poorer prognosis in previous studies [30,31].

In this study, the decision for rHCC patients to undergo re-resection was primarily based on comprehensive evaluations during MDT meetings, considering factors such as recurrence type, liver functional reserve, and tumor characteristics. Recurrence type was a key criterion, with IM typically associated with higher invasiveness and malignancy, while MO, representing de novo lesions with better prognosis, was more likely to be treated surgically. However, the determination of recurrence type in clinical practice lacks absolute standards and often relies on subjective judgment during MDT discussions, making it challenging to quantify in survival analysis. We believe that further research may clarify its role in surgical decision-making. Liver functional reserve was assessed using the Child-Pugh classification, with only grades A and B patients eligible for surgery to minimize postoperative complications. Furthermore, Child-Pugh classification was shown in our survival analysis to be closely associated with prognosis, further supporting its importance as a preoperative evaluation criterion. In terms of tumor characteristics, patients with single lesions or smaller tumor diameters were more suitable for surgical resection, while those with multiple lesions or significant extrahepatic metastases were more likely to undergo non-surgical treatments, such as TACE or systemic therapy. Additionally, factors such as ECOG score, postoperative compliance, and surgical risks were comprehensively evaluated. Patients who died within one month postoperatively were excluded from the analysis, as such deaths were primarily related to poor preoperative conditions or postoperative complications, reflecting surgical risks rather than long-term prognosis. Therefore, we focused on long-term survival to more accurately evaluate the therapeutic efficacy of re-resection. Including patients with early postoperative mortality could compromise the robustness of the model and introduce bias, particularly in short-term survival rates. These selection criteria provided a foundation for identifying surgical candidates and may offer valuable guidance for future studies.

In recent years, clinical prediction models for the diagnosis and prognosis of rHCC have been continuously updated, representing a future trend in clinical diagnosis and treatment. Zou et al. constructed recurrence prediction nomograms based on clinicopathological features such as tumor size, number, time to recurrence, HBV content, and microvascular invasion, demonstrating high predictive accuracy and reliability for rHCC prognosis after re-resection [19]. Additionally, a preoperative and postoperative ERASL model based on gender, albumin, AFP, and tumor size and number categorized the risk of HCC recurrence into high, intermediate, and low groups. This model efficiently predicted early postoperative recurrence risk and has been fully externally validated [24,32]. Our study also developed a nomogram based on prognostic risk factors in rHCC patients, aimed at identifying key factors influencing postoperative prognosis and providing a clinical tool. Due to the limited sample size, external validation was not conducted in this study. Future studies with larger sample sizes and external validation may help further assess its efficacy. Unlike existing predictive models, which primarily rely on clinical-pathological characteristics, our nomogram incorporates additional clinical factors and tumor heterogeneity, providing more comprehensive support for post-recurrence treatment decisions.

The pathogenesis of HCC involves interactions between aberrant signaling, key gene mutations, epigenetic regulation, and immune tolerance induction [33,34]. This suggests differences in molecular and immune microenvironmental typing of rHCC from different origins. Previous studies have shown that different types of recurrence (IM or MO) significantly impact the prognosis of rHCC [10,11,35]. However, research is still lacking on differentiating the two from deeper perspectives (e.g., gene mutation and immune microenvironment) and identifying relevant biosignatures that affect the prognosis of rHCC. Previous studies classified HCC into immune-activated, immune-depleted, and immune-exempt types based on the expression of immune-related genes in the tumor microenvironment, each accounting for 10 % to 25 % of HCC patients [36]. A study by Zhou et al. found a high mutation rate in proliferation-associated genes (e.g., WNK2, CTNNB1, TP53, and ARID1A) in patients with IM-type rHCC, suggesting these mutations may predict poor prognosis [37].In our study, TP53 was the most frequently mutated gene in rHCC and was associated with a higher risk of recurrence, consistent with results reported by Xue et al. [38]. A clinical prediction model incorporating TP53 mutation status may more accurately guide the classification and treatment of rHCC. Additionally, since MO-type patients are more likely to have chronic liver disease, we hypothesized differences in the immune microenvironment between early and late recurrence cohorts. We carefully selected two groups of well-preserved specimens and conducted in-depth immune microenvironmental testing. Our study found a significant increase in M2-type macrophage expression in the late recurrence group compared to the early recurrence group. M2-type macrophages, also known as alternatively activated macrophages, are widely known for their pro-tumorigenic properties and play a key role in promoting immune escape from tumors [39,40]. M2-type macrophages achieve immune escape by suppressing T cell and other immune cell activation, reducing immune surveillance of tumor cells, which is usually associated with a poorer prognosis [41,42]. However, our study showed that M2-type macrophages were more common in the advanced relapse group with better prognosis, suggesting that this group may respond better to immunotherapy, which needs further verification in future studies.

However, there are several limitations in this study. First, the clinical cases included were based on retrospective data from a single center, which may introduce selection bias and limit the generalizability of the results. Second, the sample size for gene analysis, consisting of 16 patients, is relatively small, which may lead to statistical bias and does not fully capture the molecular characteristics of rHCC. Additionally, the predictive model developed based on clinical and pathological features needs further optimization and was not externally validated due to sample size limitations. In the future, large-scale prospective clinical trials are needed to deeply investigate the molecular and immune microenvironmental features of rHCC using sophisticated methods such as single-cell sequencing. By combining clinical and pathological features with tumor molecular and immune microenvironmental characteristics, more powerful prognostic models can be developed. This will allow accurate stratification of rHCC cases, identification of patients most suitable for re-resection, and promotion of personalized therapy development.

## Conclusions

Re-resection should be considered as a potential treatment option for rHCC, associated with improved DFS and OS, comparable to initial hepatectomy. Key prognostic factors for post-re-resection outcomes include Child-Pugh classification, time to recurrence, tumor differentiation, and satellite nodules. TP53 mutations are common in rHCC and may be associated with poor prognosis. Differences in the immune microenvironment were observed between early and late recurrence groups, with higher M2 macrophage expression in the latter, suggesting potential benefit from immunotherapy. However, this hypothesis remains exploratory and requires further validation in future studies. Future prediction models should integrate clinicopathological and molecular features to accurately stratify rHCC patients and guide personalized treatment strategies.

## CRediT authorship contribution statement

**Qi Fan:** Writing – original draft, Formal analysis, Data curation. **Pengcheng Wei:** Writing – original draft, Formal analysis, Data curation. **Delin Ma:** Writing – original draft, Methodology. **Qian Cheng:** Writing – original draft, Methodology. **Jie Gao:** Writing – review & editing, Supervision, Conceptualization. **Jiye Zhu:** Writing – review & editing, Supervision, Conceptualization. **Zhao Li:** Writing – review & editing, Supervision, Funding acquisition, Conceptualization.

## Ethical approval

This study was conducted in accordance with the principles outlined in the Declaration of Helsinki. Ethical approval was obtained from the Institutional Review Board of Peking University People's Hospital (Approval No. 2022PHB092-001). Written informed consent was obtained from all participants prior to surgery, allowing the use of their clinical specimens for scientific research.

## Funding

This work was supported by the 10.13039/501100010270Capital Health Research and Development of Special Fund (2022-2-4084).

## Declaration of competing interest

No benefits in any form have been received or will be received from a commercial party related directly or indirectly to the subject of this article.
